# Development and Validation of a Nomogram to Predict Lymph Node Metastasis in Patients With T1 High-Grade Urothelial Carcinoma of the Bladder

**DOI:** 10.3389/fonc.2020.532924

**Published:** 2020-10-02

**Authors:** Ningjing Ou, Yuxuan Song, Mohan Liu, Jun Zhu, Yongjiao Yang, Xiaoqiang Liu

**Affiliations:** ^1^Department of Urology, Tianjin Medical University General Hospital, Tianjin, China; ^2^State Key Laboratory of Biotherapy and Cancer Center, West China Hospital, Sichuan University and Collaborative Innovation Center, Chengdu, China; ^3^Department of Urology, The Second Hospital of Tianjin Medical University, Tianjin, China

**Keywords:** nomogram, lymph node metastasis, MLR, bladder cancer, T1 high grade urothelial carcinoma

## Abstract

**Purpose:**

This study aims to develop and validate a nomogram to predict lymph node (LN) metastasis preoperatively in patients with T1 high-grade urothelial carcinoma.

**Methods:**

We retrospectively evaluated the data of 2,689 patients with urothelial carcinoma of the bladder (UCB) treated with radical cystectomy (RC) and bilateral lymphadenectomy in two medical centers. Eventually, 412 patients with T1 high-grade urothelial carcinoma were enrolled in the primary cohort to develop a prognostic nomogram designed to predict LN status. An independent validation cohort (containing 783 consecutive patients during the same period) was subjected to validate the predicting model. Binary regression analysis was used to develop the predicting nomogram. We assessed the performance of the nomogram concerning its clinical usefulness, calibration, and discrimination.

**Results:**

Overall, 69 (16.75%), and 135 (17.24%) patients had LN metastasis in the primary cohort and external validation cohort, respectively. The final nomogram included information on tumor number, tumor size, lymphovascular invasion (LVI), fibrinogen, and monocyte-to-lymphocyte ratio (MLR). The nomogram showed good predictive accuracy and calibration with a concordance index in the primary cohort of 0.853. The application of the nomogram in the external validation cohort still gave good discrimination (C-index, 0.845) and good calibration. The analysis of the decision curve shows that the nomogram has clinical application value.

**Conclusion:**

The nomogram that incorporated the tumor number, tumor size, LVI, fibrinogen, and MLR showed favorable predictive accuracy for LN metastasis. It may be conveniently used to predict LN metastasis in patients with T1 high-grade urothelial carcinoma and be helpful in guiding treatment decisions.

## Introduction

Urothelial carcinoma of the bladder (UCB) is the ninth most common tumor worldwide (1). It is also the most prevalent cancer of the urinary tract and the fourth cause of cancer-related death in men (2). Approximately 75% of patients with UCB are diagnosed as non-muscle-invasive bladder cancer (NMIBC) at the first time in seeking medical care. Most of the NMIBC patients will choose to retain bladder therapy: transurethral resection of the bladder (TURBT) combined with maintenance bacillus Calmette–Guérin (BCG), but some patients (especially T1 high grade) are very likely to fail BCG therapy alone (3). Approximately 22% of the UCB patients finally found positive lymph nodes, and this number will increase with pathologic stage progressing (13% for pT1 and 45% for pT4). To date, extended pelvic lymph node (LN) dissection is suggested in some clinical trials for UCB patients with LN metastasis. It can prolong the lifetime of these patients (4, 5). Immediate radical cystectomy (RC) and LN dissection should be considered in selected cases such as those patients with pT1 high-grade disease and other risk factors such as lymphovascular invasion (LVI), histological variants, and size of the tumor (6). However, some patients with T1 high-grade bladder cancer have a low risk of metastasis and progression, and conservative treatment can be tried. In these cases, RC may be an overtreatment (7). Some researchers found that even muscle-invasive bladder tumor can be treated with trigeminal therapy that can preserve the bladder and improve the quality of the patient’s life (8). At the same time, some scholars believe that extended lymph dissection should be performed during RC for patients with higher risk of LN metastasis. However, others thought that the extent of pelvic LN dissection (PLND) of T1 high-grade UCB remains highly disputed if there has no evidence of LN metastasis (9–11). A good preoperative prediction about LN metastasis can be helpful in making a decision on PLND (10, 12). There is no validated project to decide which treatment program is optimal for each patient with T1 high-grade disease. Current evidence demonstrate that clinicopathological and molecular risk classifiers together may help to select the optimal management strategy for each patient with T1 high-grade disease (7).

Recently, several researches have found that systemic inflammatory response is highly related to LN metastasis and tumor progression (13, 14). Some studies have found that preoperative neutrophil-to-lymphocyte ratio (NLR), platelet-to-lymphocyte ratio (PLR), monocyte-to-lymphocyte ratio (MLR), and fibrinogen are directly associated with nodal involvement status of tumors (15–19). Kilian et al. found that decreased cholinesterase (ChE) is associated with poor prognosis in patients with NMIBC undergoing TURBT (8). Ferro et al. found that type 2 diabetes mellitus predicts worse outcomes in patients with high-grade T1 bladder cancer (20). These results indicate that many routine laboratory reports will be helpful to predict the prognosis of tumors.

Hence, this study was aimed to detect the relationship between inflammation biomarkers, clinicopathological features, and LN metastasis in patients with T1 high-grade UCB treated with RC. This study also aimed to develop and validate a nomogram to predict LN metastasis preoperatively in patients with T1 high-grade urothelial carcinoma.

## Materials and Methods

**FIGURE 1:**
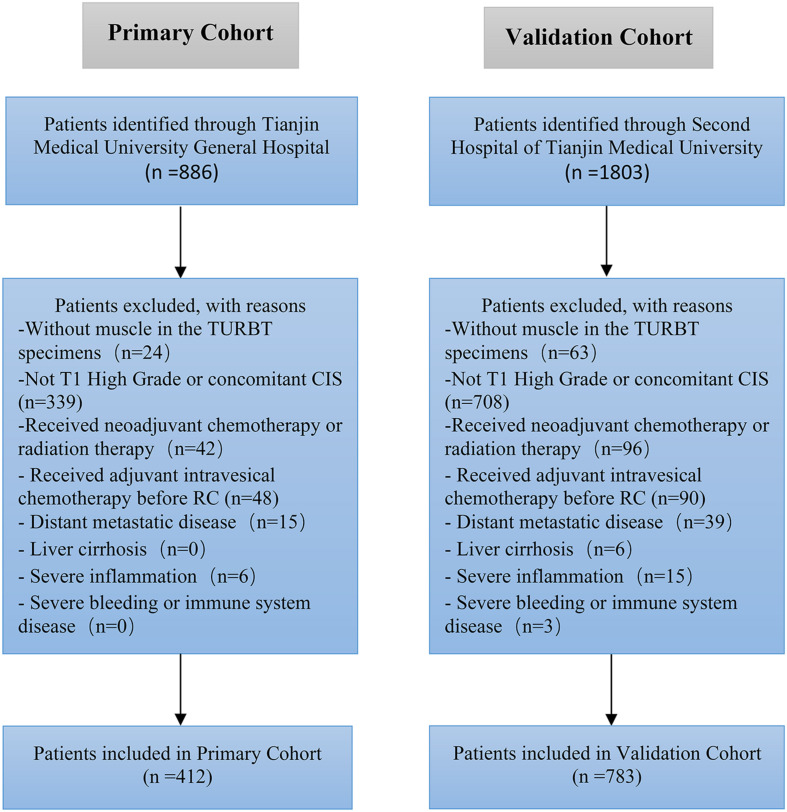
Flow chart of the enrollment of patients.

We retrospectively evaluated the data of 2,689 patients with UCB treated with RC and bilateral lymphadenectomy at the Second Hospital of Tianjin Medical University and Tianjin Medical University General Hospital between January 2010 and December 2019. Eventually, we enrolled 1,195 patients (412 in the primary cohort and 783 in the validation cohort) with T1 high-grade UCB in this study (Figure 1). All patients enrolled had RC immediately (no more than 1 month) after histologically confirmed pT1 high-grade disease. Approximately 35% of the patients had repeated TURBT. The tumor was graded according to the World Health Organization—International Society of Urologic Pathology 2004 guidelines and the tumor node metastasis (TNM) 2002 staging system. All patients were histologically confirmed, and the exclusion criteria were as follows: (a) patients without muscle in the TURBT specimen, (b) patients received radiation therapy or neoadjuvant chemotherapy, (c) liver cirrhosis, (d) patients had distant metastatic disease at the time of RC, (e) severe inflammation, (f) immune system or severe bleeding disease, (g) patients had concomitant carcinoma *in situ* (CIS), and (h) patients received adjuvant intravesical chemo before RC.

Sex, age, lymphatic invasion, LN status, tumor number, tumor size, TNM stage, and routine preoperative laboratory measurements including white blood cell (WBC) count, neutrophil count, lymphocyte count, monocyte count, hemoglobin (HB), albumin (ALB), and fibrinogen were recorded in our study. Two doctors independently reviewed the medical data and pathological data of all patients.

### PLR, NLR, and MLR Evaluation

Blood count test was a routine part of the preoperative inspection before the surgery. All blood samples were obtained within 14 days (median 6.3 days) before RC. The definition of PLR, NLR, and MLR was platelet count divided by lymphocyte count, neutrophil count divided by lymphocyte count, and monocyte count divided by lymphocyte count, respectively.

### Statistical Analysis

Our patients were divided into two groups on the basis of LN status. Continuous data were presented as means ± standard deviations. Mann–Whitney *U*-test was conducted for all variables with non-normal distribution. The features of clinicopathological and categorical variables associated with LN status were analyzed using the χ2 test. Statistical analyses were performed using IBM SPSS (version 22.0) and R software (version 3.5.2). *P* < 0.05 was accepted as statistically significant.

### Development of Prediction Model

Binary logistic regression analysis was conducted with the following clinical candidate predictors: tumor number, tumor size, LVI, fibrinogen, NLR, PLR, and MLR. Then, tumor number, tumor size, LVI, fibrinogen, and MLR were applied to develop a diagnostic model for LN metastasis by using the primary cohort. Backward step-wise selection was applied by using the likelihood ratio test with Akaike’s information criterion as the stopping rule to develop and validate the nomogram.

### Performance of the Nomogram in the Primary Cohort

Calibration curves and Hosmer–Lemeshow test were conducted to assess the calibration of the nomogram. To quantify the discrimination performance of the nomogram, Harrell’s C-index was used. Bootstrapping validation (1,000 bootstrap resample) was used to calculate a relatively corrected C-index.

### Validation of the Nomogram

Internal validation was performed. The logistic regression formula was built with the primary cohort and was applied to the validation cohort, with total points for each patient evaluated. The total points were then subjected to logistic regression as a factor. Decision curve analysis was performed to assess the clinical usefulness of the nomogram.

## Results

### Characteristics

Patients’ characteristic of the primary cohort and validation cohort showed in Table 1. No significant difference was found between primary cohort (16.75%, 69/412) and validation cohort (17.24%, 135/783) in regard to lymph node-positive rate (*P* = 0.462). Patients with LN metastasis in both primary and validation cohorts were more likely to have bigger tumor (≥3 cm), multiple tumors, hydronephrosis, and LVI than the patients without LN metastasis. There is no significant difference association with LN status for other parameters, such as gender, age, body mass index (BMI), and ALB.

**TABLE 1:** Clinicopathologic features of patients according to the lymph node status.

Parameters	Primary cohort				Validation cohort			
	Overall *n* = 412	LN (+) *n* = 69	LN (−) *n* = 343	*P*	Overall *n* = 783	LN (+) *n* = 135	LN (−) *n* = 648	*P*
**Gender**				0.481				0.189
Male	372	63(16.9)	309(83.1)		487	123(25.3)	564(74.7)	
Female	40	6(15.0)	34(85.0)		96	12(12.5)	84(87.5)	
**Age (years)**				0.184				0.526
≥60	264	48(18.2)	216(77.8)		574	96(16.7)	478(83.3)	
<60	148	21(14.2)	127(85.8)		209	39(18.7)	170(80.3)	
**BMI**				0.143				0.273
≥28	45	11(24.4)	34(65.6)		69	18(26.1)	51 (73.9)	
<28	367	58(15.8)	309(84.2)		728	149(20.4)	579(79.6)	
**Hydronephrosis**				0.011				0.001
Present	64	18(28.1)	47(61.9)		114	36(31.6)	78(68.4)	
Absent	347	51(14.7)	296(85.3)		669	99(14.8)	570(85.2)	
ALB(g/l)				0.125				0.189
≥35	324	49(15.1)	275(84.9)		624	102(16.3)	522(83.7)	
<35	88	20(22.2)	68(77.8)		159	33(20.8)	126(79.2)	
**Pathological stage**				0.001*				<0.001*
pTa	21	0(0.0)	21(100.0)		27	0(0.00)	27(100.0)	
pT1	98	9(9.2)	89(90.8)		337	39(11.6)	288(88.4)	
pT2	149	18(12.1)	131(87.9)		343	39(11.3)	204(88.7)	
pT3	101	21(20.1)	80(79.9)		121	33(27.3)	88(72.7)	
pT4	39	18(46.2)	21(53.8)		65	24(36.9)	41(63.1)	
≥pT1	293	60(20.5)	233(79.5)		429	96(22.3)	333(77.5)	
**LVI, no. (%)**				<0.001				<0.001
Present	135	42(38.9)	93(61.1)		303	99(32.7)	204(67.3)	
Absent	277	27(13.2)	250(86.8)		480	36(7.5)	444(92.5)	
**Tumor size (cm)**				<0.001				<0.001
≥3	114	42(36.8)	72(53.2)		364	99(27.2)	265(72.7)	
<3	341	27(7.9)	271(92.1)		419	36(8.5)	383(91.5)	
**Tumor number**				<0.001				0.012
Multiple	174	51(29.3)	123(70.7)		362	87(24.0)	275(76.0)	
Single	264	18(6.8)	246(93.2)		421	48(11.4)	373(88.6)	

BMI, body mass index; ALB, albumin; and LVI, lymphovascular invasion. *Chi-square tests of pT1 and ≥pT1.

### Features of Preoperative Inflammatory Indicators

As shown in Table 2, although the WBC count did not show any difference between the two groups (*P* = 0.393) concerning the nodal status, fibrinogen (*P* < 0.001), and lymphocyte count (*P* < 0.001) were significantly higher in patients with nodal involvement than those without. The differences between the lymph node-positive and lymph node-negative groups were statistically significant in regard to HB (*P* = 0.009), PLR (*P* = 0.019), NLR (*P* = 0.033), and MLR (*P* = 0.012). Other parameters like neutrophil count, monocyte count, platelet count, and ALB did not show an association with LN status.

**TABLE 2:** Inflammation indicators according to lymph node status.

Parameters	Primary cohort			Validation cohort		
	LN (+)	LN (−)	*P*	LN (+)	LN (−)	*P*
WBC count	6.78 ± 1.92	7.31 ± 4.17	0.393	7.47 ± 1.99	7.00 ± 1.68	0.311
Neutrophil count	4.67 ± 1.76	4.60 ± 178	0.792	5.15 ± 1.85	4.53 ± 1.54	0.153
Lymphocyte count	1.42 ± 0.66	1.82 ± 0.66	<0.001	1.34 ± 0.71	1.82 ± 0.58	0.006
Monocyte count	0.42 ± 0.16	0.44 ± 0.17	0.523	0.49 ± 0.16	0.45 ± 0.20	0.395
Platelet count	229.38 ± 66.32	237.50 ± 71.24	0.486	233.09 ± 40.03	254.60 ± 65.916	0.140
HB	124.58 ± 21.47	133.58 ± 20.38	0.009	123.61 ± 20.71	133.10 ± 22.53	0.073
ALB	38.91 ± 6.30	39.58 ± 6.22	0.522	37.09 ± 7.00	39.24 ± 6.69	0.198
Fibrinogen	3.87 ± 0.76	3.36 ± 0.87	<0.001	4.25 ± 0.76	3.35 ± 0.86	0.001
NLR	3.98 ± 3.09	3.01 ± 2.65	0.033	4.67 ± 2.29	2.81 ± 1.42	0.001
PLR	193.85 ± 119.73	148.40 ± 80.30	0.019	248.92 ± 167.54	157.37 ± 73.53	0.017
MLR	0.34 ± 0.15	0.27 ± 0.14	0.012	0.48 ± 0.24	0.28 ± 0.17	0.001

*WBC, white blood cell; HB, hemoglobin; ALB, albumin; NLR, neutrophil-to-lymphocyte ratio; PLR, platelet-to-lymphocyte ratio; and MLR, monocyte-to-lymphocyte ratio.*

### Development of the Prediction Model

**FIGURE 2:**
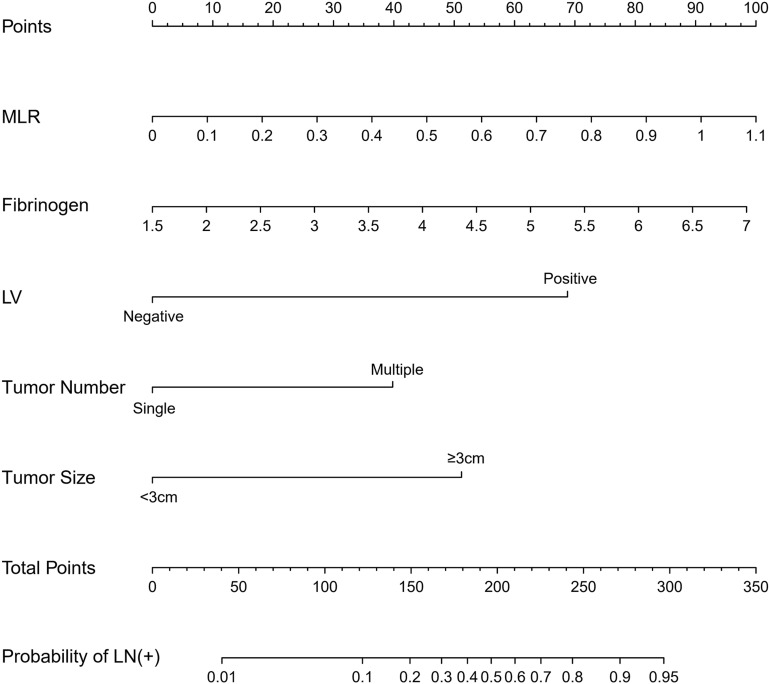
The nomogram was developed in the primary cohort, with tumor number, tumor size, lymphovascular invasion, fibrinogen, and MLR.

Binary regression analysis identified tumor number, tumor size, LVI, fibrinogen, and MLR as independent predictors (Table 3). These independent predictors were pooled to develop the prediction model. The prediction model was demonstrated as a nomogram (Figure 2).

**TABLE 3:** Risk factors for LN metastasis.

Variable and intercept	Univariate logistic regression		Multivariate logistic regression	
	Adjusted OR (95% CI)	*P*	Adjusted OR (95% CI)	*P*
Hydronephrosis	2.66(1.20−5.86)	0.016		
Lymphocyte count	0.35(0.19−0.66)	0.01		
HB	0.98(0.965−1.00)	0.10		
Fibrinogen	1.89(1.31−2.73)	<0.001	1.88 (1.18–2.99)	0.008
NLR	1.12(0.99−1.24)	0.074		
PLR	1.004(0.961−1.008)	0.236		
MLR	5.81(1.69−52.13)	0.007	7.79 (1.94–60.06)	0.003
Lymphovascular invasion	6.00(2.89−12.43)	<0.001	8.50 (3.66–19.74)	<0.001
Tumor size	4.28(2.19−8.36)	<0.001	4.71 (2.06–10.78)	<0.001
Tumor number	2.8(1.5−5.24)	0.001	3.50 (1.53–8.06)	0.001

*NLR, neutrophil-to-lymphocyte ratio; PLR, platelet-to-lymphocyte ratio; and MLR, monocyte-to-lymphocyte ratio.*

### Performance of the Nomogram

**FIGURE 3:**
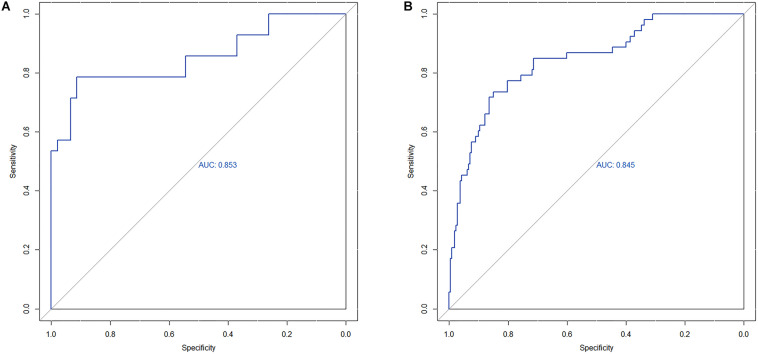
**(A)** The ROC curve of nomogram in the primary cohort. **(B)** The ROC curve of nomogram in the validation cohort.

**FIGURE 4:**
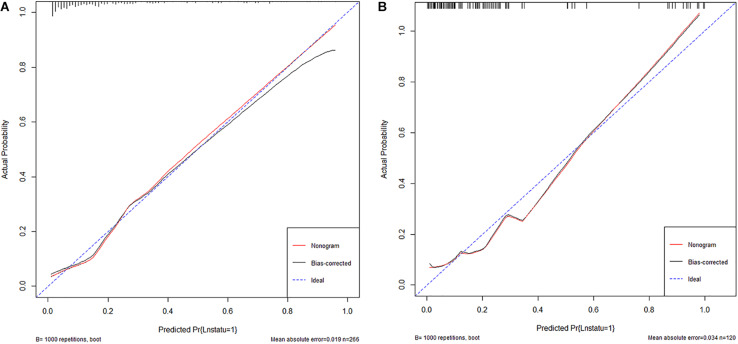
**(A)** The calibration curve of nomogram in the primary cohort. **(B)** The calibration curve of nomogram in the validation cohort.

The area under the curve (AUC) of the receiver operating characteristic (ROC) curve (Figure 3) of the nomogram was 0.853 (95% CI, 0.785 to 0.906), indicating that the nomogram is powerful to differentiate LN metastasis. The calibration curve of the nomogram demonstrated a good agreement between the observation cases and prediction cases both in the primary cohort and validation cohort (Figure 4). The Hosmer–Lemeshow test of the nomogram harvested a non-significant statistic in the primary cohort (*P* = 0.381) and validation cohort (*P* = 0.236). The C-index of the nomogram was 0.853 (95% CI, 0.77 to 0.91) in the primary cohort, which was confirmed to be 0.845 (95% CI, 0.76 to 0.98) in the validation cohort.

### Clinical Use

**FIGURE 5:**
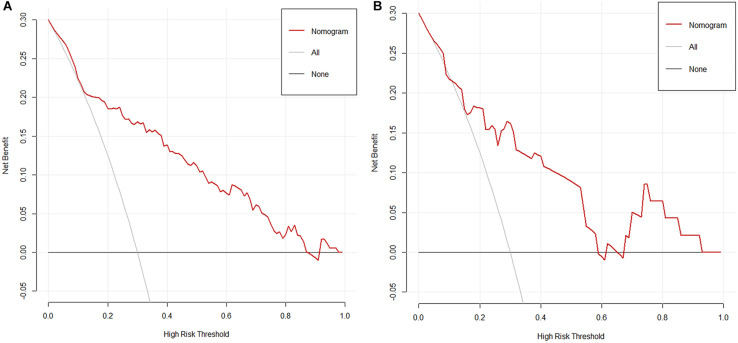
**(A)** The decision curve of nomogram in the primary cohort. **(B)** The decision curve of nomogram in the validation cohort. Net benefit can be interpreted as the proportion of all patients who have lymph node metastases and are recommended for extended pelvic lymph node dissection if no patients with negative lymph nodes were treated.

The result of the decision curve (Figure 5) in regard to the primary cohort demonstrated that when the threshold probability was 0.15–0.80, the nomogram will be helpful to predict LN metastases. Correspondingly, if the threshold probability was 0.20–0.60 in the validation cohort, using the nomogram to predict LN metastases and make treatment decision adds more benefit than either the treat-all-patients scheme or the treat-none scheme.

## Discussion

As is known to all, cancer-specific survival (CSS) and overall survival (OS) of patients with UCB were associated with pathologic stage and LN status at the time of cystectomy. The potential of LN metastasis plays an important role when considering an operation (21). Fritsche et al. reported that 16.2% of the patients treated with RC for T1 high-grade UCB had LN metastasis (22). If we can identify patients who may have LN metastasis with T1 high-grade UCB before surgical treatment, it will facilitate individualized treatment of the disease.

Nowadays, plenty of methods including quantitative comparative proteomic, imaging techniques (such as PET-CT and MRI) (23, 24), and histological analysis have been used to identify new markers predicting LN metastasis. Brunocilla et al. evaluated the role of PET-CT in the preoperative evaluation of the nodal involvement of patients with BC suitable for RC and found that PET-CT showed a sensitivity of 42% and a specificity of 84%, whereas contrast-enhanced CT showed a sensitivity of 14% and a specificity of 89% (24). Birkhäuser et al. assessed the diagnostic accuracy of combined ultrasmall superparamagnetic particles of iron oxide (USPIO) MRI and diffusion-weighted (DW) MRI in staging of normal-sized pelvic LNs in bladder cancer patients. The results showed that per-patient sensitivity and specificity for detection of LN metastases by the three readers ranged from 65% to 75% and 93% to 96%, respectively (25). All of the above results indicate that it is difficult to predict LN metastasis accurately even using the most advanced imaging techniques. Therefore, we wanted to explore a more accurate model to predict LN metastasis of T1 high-grade BC by combining the pathological characteristics of TURBT specimens with inflammation indicators in peripheral blood. Cantiello et al. investigated 1,151 NMIBC patients who underwent first TURBT at 13 academic institutions and found that NLR, PLR, and LMR are factors predictive of recurrence, progression, cancer-specific mortality, and overall mortality (26). Previous studies have demonstrated that NLR, PLR, and fibrinogen are available biomarkers to predict LN metastasis (21, 27), and other hematological indicators, like MLR and ALB, have been shown to be associated with pathologic stage or LN status of bladder carcinoma (17, 27–29). A study involving patients with high-grade T1 bladder cancer also showed that absolute basophil count is associated with time to recurrence in patients receiving BCG after TURBT tumor (30). Hematological indicators are cheap and accessible. However, their clinical applications are unrealistic because there is no good sensitivity and specificity to use them solely.

In our study, we found LN metastasis in about 20% of the patients who underwent RC for T1 high-grade UCB. It is higher than the previous study conducted by Fritsche et al. We suspect that the difference may come from the different ethnicity of patients or may have resulted from the different treatment strategies (our patients received less repeated TURBT and adjuvant intravesical chemotherapy before RC). In our results, there is no significant difference in lymphocyte and monocyte counts between the two groups, but both lymphocyte count and monocyte count were increased in the lymph node-positive group. Monocytes and tumor-associated macrophages (differentiated by monocytes) were thought to have an impact on tumorigenesis and will be recruited to the tumor site (31). The main function of lymphocytes is suppressing proliferation and migration of cancer cell (32). Therefore, monocyte and lymphocyte count (to suppress migration of cancer cells) may increase or decrease in patients having tumor. Besides, after calculating the ratio of platelet count, neutrophil count, and monocyte count to lymphocyte count, we found that NLR, PLR, and MLR were significantly correlated with LN metastasis in bladder tumors, which is consistent with previous studies.

According to our results, there were significant differences about the means of PLR, NLR, MLR, and fibrinogen between the lymph node-positive and lymph node-negative groups. Also, the tumor number, tumor size, LVI, fibrinogen, and MLR, according to the logistic regression analysis, are independent predictors of LN metastasis in patients with T1 high-grade urothelial carcinoma. It suggests that there is a close relationship between cancer and inflammation (33).

Pathologic features, such as tumor number, tumor size, and LVI are widely accepted as an indicator of tumor prognosis. Thus, we attempted to develop a model including tumor number, tumor size, LVI, fibrinogen, PLR, NLR, and MLR for the prediction of LN metastasis. Then, we found that the tumor number, tumor size, LVI, fibrinogen, and MLR are independent predictors of LN metastasis in patients with T1 high-grade urothelial carcinoma. Our results are consistent with previous research. Ferro et al. found that tumor size and the presence of carcinoma *in situ* are independent predictors to identify patients at risk of residual T1 high-grade bladder cancer after a complete TUR (34). This suggests that the combination of pathological features and inflammatory status is a good predictor of LN metastasis in patients with T1 high-grade urothelial carcinoma (12).

Therefore, we developed a nomogram of tumor number, tumor size, LVI, fibrinogen, and MLR to help doctors and patients make better decisions on the treatment of T1 high-grade urothelial carcinoma. The Hosmer–Leme show test of the nomogram shows that this model has a good fit. The nomogram of this combination was conducted to present it. The AUC (0.843) of the ROC curve of the nomogram indicated that the nomogram is powerful to differentiate LN metastasis. The calibration curve demonstrated good consistency in the primary cohort and validation cohort. To justify the clinical usefulness of the nomogram, we assessed whether nomogram-assisted decisions would improve patient outcomes. The decision curve showed that the nomogram will be helpful to predict LN metastases. All of the measurements above indicate that the nomogram of tumor number, tumor size, LVI, fibrinogen, and MLR can help doctors and patients make better decisions on the treatment of T1 high-grade urothelial carcinoma.

Nevertheless, our study has many limitations. First, even if we collected two institutions’ data, the sample size remains small. More and more patients with T1 high-grade bladder tumor choose to retain the bladder, and fewer and fewer patients receive RC in the early stage. We believe that even if we wait a few more years, the number of cases will not increase significantly. However, considering that 20% of the patients will still benefit from the early standard full cut, we believe that more patients will benefit from using this model to assist in clinical decision making. Second, although we have tried our best to control potential confounders, we could not control surgeon and pathologist experience, treatment decisions, including patient and surgeon preferences. Fortunately, we have two pathologists to review to identify LNs. Third, the data were collected retrospectively. However, for now, the model was developed by the best data we can achieve, and it may provide some reference for clinical decision making. In future studies, we will design a prospective randomized controlled study with a higher level of evidence to further validate our model.

## Conclusion

The nomogram that incorporated the tumor number, tumor size, LVI, fibrinogen, and MLR showed favorable predictive accuracy for LN metastasis. It may be conveniently used to predict LN metastasis in patients with T1 high-grade urothelial carcinoma and be helpful in guiding treatment decisions. Large-sample multicenter validation is needed for its clinical application.

## Data Availability Statement

The datasets generated for this study are available on request to the corresponding author.

## Ethics Statement

The study protocol was approved by the internal research Ethics Committee of Tianjin Medical University General Hospital. The patients/participants provided their written informed consent to participate in this study.

## Author Contributions

The project was designed by XL and JZ. YS and ML congregated data and analyzed it. YY and NO wrote the manuscript. All authors have read and approved the final manuscript.

## Conflict of Interest

The authors declare that the research was conducted in the absence of any commercial or financial relationships that could be construed as a potential conflict of interest.

## Abbreviations

LN: lymph node
UCB: urothelial carcinoma of the bladder
NLR: neutrophil-to-lymphocyte ratio
PLR: platelet-to-lymphocyte ratio
MLR: monocyte-to-lymphocyte ratio
NMIBC: non-muscle invasive bladder cancer
PLND: pelvic LN dissection
ChE: cholinesterase
TURBT: transurethral resection of the bladder
RC: radical cystectomy
TNM: tumor node metastasis
HB: hemoglobin
ALB: albumin
WBC: white blood cell
AUC: area under the curve
CSS: cancer-specific survival
OS: overall survival.

## References

[1] Antoni S Ferlay J Soerjomataram I Znaor A Jemal A Bray F. Bladder cancer incidence and mortality: a global overview and recent trends. *Eur Urol.* (2017) 71:96–108. 10.1016/j.eururo.2016.06.010 27370177

[2] Siegel R Ma J Zou Z Jemal A. Cancer statistics, 2014. *CA Cancer J Clin.* (2014) 64:9–29. 10.3322/caac.21208 24399786

[3] Ferro M Vartolomei MD Cantiello F Lucarelli G Di Stasi SM Hurle R High-grade T1 on re-transurethral resection after initial high-grade T1 confers worse oncological outcomes: results of a multi-institutional study. *Urol Int.* (2018) 101:7–15. 10.1159/000490765 29975950

[4] Karl A Carroll PR Gschwend JE Knuchel R Montorsi F Stief CG The impact of lymphadenectomy and lymph node metastasis on the outcomes of radical cystectomy for bladder cancer. *Eur Urol.* (2009) 55:826–35. 10.1016/j.eururo.2009.01.004 19150582

[5] Ghodoussipour S Daneshmand S. Current controversies on the role of lymphadenectomy for bladder cancer. *Urol Oncol.* (2019) 37:193–200. 10.1016/j.urolonc.2018.05.005 29909945

[6] Babjuk M Bohle A Burger M Capoun O Cohen D Comperat EM EAU guidelines on non-muscle-invasive urothelial carcinoma of the bladder: update 2016. *Eur Urol.* (2017) 71:447–61. 10.1016/j.eururo.2016.05.041 27324428

[7] Yun SJ Kim SK Kim WJ. How do we manage high-grade T1 bladder cancer? Conservative or aggressive therapy? *Investig Clin Urol.* (2016) 57(Suppl. 1):S44–51. 10.4111/icu.2016.57.S1.S44 27326407 PMC4910762

[8] Royce TJ Feldman AS Mossanen M Yang JC Shipley WU Pandharipande PV Comparative effectiveness of bladder-preserving tri-modality therapy versus radical cystectomy for muscle-invasive bladder cancer. *Clin Genitourin Cancer.* (2019) 17:23–31.e3. 10.1016/j.clgc.2018.09.023 30482661

[9] Zehnder P Studer UE Daneshmand S Birkhauser FD Skinner EC Roth B Outcomes of radical cystectomy with extended lymphadenectomy alone in patients with lymph node-positive bladder cancer who are unfit for or who decline adjuvant chemotherapy. *BJU Int.* (2014) 113:554–60. 10.1111/bju.12520 24131453

[10] Zargar-Shoshtari K Zargar H Lotan Y Shah JB van Rhijn BW Daneshmand S A multi-institutional analysis of outcomes of patients with clinically node positive urothelial bladder cancer treated with induction chemotherapy and radical cystectomy. *J Urol.* (2016) 195:53–9. 10.1016/j.juro.2015.07.085 26205531

[11] Shariat SF Karakiewicz PI Palapattu GS Lotan Y Rogers CG Amiel GE Outcomes of radical cystectomy for transitional cell carcinoma of the bladder: a contemporary series from the Bladder Cancer Research Consortium. *J Urol.* (2006) 176:2414–22; discussion 22. 10.1016/j.juro.2006.08.004 17085118

[12] Kluth LA Black PC Bochner BH Catto J Lerner SP Stenzl A Prognostic and prediction tools in bladder cancer: a comprehensive review of the literature. *Eur Urol.* (2015) 68:238–53. 10.1016/j.eururo.2015.01.032 25709027

[13] Balkwill F Mantovani A. Inflammation and cancer: back to Virchow? *Lancet.* (2001) 357:539–45. 10.1016/S0140-6736(00)04046-0 11229684

[14] Shariat SF Rink M Ehdaie B Xylinas E Babjuk M Merseburger AS Pathologic nodal staging score for bladder cancer: a decision tool for adjuvant therapy after radical cystectomy. *Eur Urol.* (2013) 63:371–8. 10.1016/j.eururo.2012.06.008 22727174

[15] Yoshida T Kinoshita H Yoshida K Mishima T Yanishi M Inui H Prognostic impact of perioperative lymphocyte-monocyte ratio in patients with bladder cancer undergoing radical cystectomy. *Tumour Biol.* (2016) 37:10067–74. 10.1007/s13277-016-4874-8 26819209

[16] Ma C Lu B Diao C Zhao K Wang X Ma B Preoperative neutrophil-lymphocyte ratio and fibrinogen level in patients distinguish between muscle-invasive bladder cancer and non-muscle-invasive bladder cancer. *Onco Targets Ther.* (2016) 9:4917–22. 10.2147/OTT.S107445 27540305 PMC4982501

[17] D’Andrea D Moschini M Gust KM Abufaraj M Ozsoy M Mathieu R Lymphocyte-to-monocyte ratio and neutrophil-to-lymphocyte ratio as biomarkers for predicting lymph node metastasis and survival in patients treated with radical cystectomy. *J Surg Oncol.* (2017) 115:455–61. 10.1002/jso.24521 28105663

[18] Vartolomei MD Ferro M Cantiello F Lucarelli G Di Stasi S Hurle R Validation of neutrophil-to-lymphocyte ratio in a multi-institutional cohort of patients with T1G3 non-muscle-invasive bladder cancer. *Clin Genitourin Cancer.* (2018) 16:445–52. 10.1016/j.clgc.2018.07.003 30077463

[19] Hermanns T Bhindi B Wei Y Yu J Noon AP Richard PO Pre-treatment neutrophil-to-lymphocyte ratio as predictor of adverse outcomes in patients undergoing radical cystectomy for urothelial carcinoma of the bladder. *Br J Cancer.* (2014) 111:444–51. 10.1038/bjc.2014.305 24918819 PMC4119979

[20] Ferro M Katalin MO Buonerba C Marian R Cantiello F Musi G Type 2 diabetes mellitus predicts worse outcomes in patients with high-grade T1 bladder cancer receiving bacillus Calmette-Guérin after transurethral resection of the bladder tumor. *Urol Oncol.* (2020) 38:459–64. 10.1016/j.urolonc.2020.02.016 32173242

[21] Pang W Lou N Jin C Hu C Arvine C Zhu G Combination of preoperative platelet/lymphocyte and neutrophil/lymphocyte rates and tumor-related factors to predict lymph node metastasis in patients with gastric cancer. *Eur J Gastroenterol Hepatol.* (2016) 28:493–502. 10.1097/MEG.0000000000000563 26854795 PMC4892768

[22] Fritsche HM Burger M Svatek RS Jeldres C Karakiewicz PI Novara G Characteristics and outcomes of patients with clinical T1 grade 3 urothelial carcinoma treated with radical cystectomy: results from an international cohort. *Eur Urol.* (2010) 57:300–9. 19766384 10.1016/j.eururo.2009.09.024

[23] Kiss B Thoeny HC Studer UE. Current status of lymph node imaging in bladder and prostate cancer. *Urology.* (2016) 96:1–7. 10.1016/j.urology.2016.02.014 26966038

[24] Brunocilla E Ceci F Schiavina R Castellucci P Maffione AM Cevenini M Diagnostic accuracy of. (11)C-choline PET/CT in preoperative lymph node staging of bladder cancer: a systematic comparison with contrast-enhanced CT and histologic findings. *Clin Nucl Med.* (2014) 39:e308–12. 10.1097/RLU.0000000000000342 24458183

[25] Birkhäuser FD Studer UE Froehlich JM Triantafyllou M Bains LJ Petralia G Combined ultrasmall superparamagnetic particles of iron oxide-enhanced and diffusion-weighted magnetic resonance imaging facilitates detection of metastases in normal-sized pelvic lymph nodes of patients with bladder and prostate cancer. *Eur Urol.* (2013) 64:953–60. 10.1016/j.eururo.2013.07.032 23916692

[26] Cantiello F Russo GI Vartolomei MD Farhan ARA Terracciano D Musi G Systemic inflammatory markers and oncologic outcomes in patients with high-risk non-muscle-invasive urothelial bladder cancer. *Eur Urol Oncol.* (2018) 1:403–10. 31158079 10.1016/j.euo.2018.06.006

[27] Xiang J Zhou L Li X Bao W Chen T Xi X Preoperative monocyte-to-lymphocyte ratio in peripheral blood predicts stages, metastasis, and histological grades in patients with ovarian cancer. *Transl Oncol.* (2017) 10:33–9. 10.1016/j.tranon.2016.10.006 27888711 PMC5124360

[28] Viers BR Boorjian SA Frank I Tarrell RF Thapa P Karnes RJ Pretreatment neutrophil-to-lymphocyte ratio is associated with advanced pathologic tumor stage and increased cancer-specific mortality among patients with urothelial carcinoma of the bladder undergoing radical cystectomy. *Eur Urol.* (2014) 66:1157–64. 10.1016/j.eururo.2014.02.042 24630414

[29] Ferro M De Cobelli O Buonerba C Di Lorenzo G Capece M Bruzzese D Modified glasgow prognostic score is associated with risk of recurrence in bladder cancer patients after radical cystectomy: a multicenter experience. *Medicine.* (2015) 94:e1861. 10.1097/MD.0000000000001861 26496339 PMC4620818

[30] Ferro M Di Lorenzo G Vartolomei MD Bruzzese D Cantiello F Lucarelli G Absolute basophil count is associated with time to recurrence in patients with high-grade T1 bladder cancer receiving bacillus Calmette-Guérin after transurethral resection of the bladder tumor. *World J Urol.* (2020) 38:143–50. 10.1007/s00345-019-02754-2 30993426

[31] Chanmee T Ontong P Konno K Itano N. Tumor-associated macrophages as major players in the tumor microenvironment. *Cancers.* (2014) 6:1670–90. 10.3390/cancers6031670 25125485 PMC4190561

[32] Bastid J Bonnefoy N Eliaou JF Bensussan A. Lymphocyte-derived interleukin-17A adds another brick in the wall of inflammation-induced breast carcinogenesis. *Oncoimmunology.* (2014) 3:e28273. 10.4161/onci.28273 25050201 PMC4063083

[33] Diakos CI Charles KA McMillan DC Clarke SJ. Cancer-related inflammation and treatment effectiveness. *Lancet Oncol.* (2014) 15:e493–503. 10.1016/S1470-2045(14)70263-3 25281468

[34] Ferro M Di Lorenzo G Buonerba C Lucarelli G Russo GI Cantiello F Predictors of residual T1 high grade on re-transurethral resection in a large multi-institutional cohort of patients with primary T1 high-grade/grade 3 Bladder cancer. *J Cancer.* (2018) 9:4250–4. 10.7150/jca.26129 30519326 PMC6277616

